# Cytotoxic and antiviral activities of *Jatropha variegata* and* Jatropha spinosa* in relation to their metabolite profile

**DOI:** 10.1038/s41598-024-55196-1

**Published:** 2024-02-28

**Authors:** Khawlah Shari, Osama G. Mohamed, Khaled M. Meselhy, Ashootosh Tripathi, Amal E. Khaleel, Essam Abdel-Sattar, Rania A. El Gedaily

**Affiliations:** 1 Pharmacognosy Department, Faculty of Pharmacy, Cairo University, Kasr El Aini St, Cairo, 11562 Egypt; 2 Natural Products Discovery Core, Life Sciences Institute, University of Michigan, Ann Arbor, MI 48109 USA; 3 Department of Medicinal Chemistry, College of Pharmacy, University of Michigan, Ann Arbor, MI 48109 USA

**Keywords:** *Jatropha variegata*, *Jatropha spinosa*, Cytotoxicity, Antiviral, UPLC/MS, Diterpenes, Cancer, Drug discovery, Plant sciences, Chemistry

## Abstract

*Jatropha variegata* and *Jatropha spinosa* (family: Euphorbiaceae) are utilized in Yemeni traditional medicine to treat respiratory tract infection and in different skin conditions such as wound healing, as antibacterial and hemostatic. In this study, we evaluated the cytotoxicity and the antiviral activities of the methanolic *J. variegata* (leaves: **Ext-1**, stems: **Ext-2**, and roots: **Ext-3**), and *J. spinosa* extracts (aerial parts: **Ext-4** and roots: **Ext-5**), in addition to their methylene chloride fractions of roots extracts (**F-6** and **F-7**, respectively). All samples were tested against three human cancer cell lines in vitro (MCF-7, HepG2, and A549) and two viruses (HSV-2 and H1N1). Both plants showed significant cytotoxicity, among them, the methylene chloride fractions of roots of *J. variegata (***F-6*)*** and *J. spinosa* roots **(F-7)** showed the highest activity on MCF-7 (IC_50_ = 1.4 and 1 μg/mL), HepG2 (IC_50_ = 0.64 and 0.24 μg/mL), and A549 (IC_50_ = 0.7 and 0.5 μg/mL), respectively, whereas the IC_50_ values of the standard doxorubicin were (3.83, 4.73, and 4.57 μg/mL) against MCF-7, HepG2, and A549, respectively. These results revealed that the roots of both plants are potential targets for cytotoxic activities. The in vitro results revealed potential antiviral activity for each of **Ext-3, Ext-5, F-6, and F-7** against HVS-2 with IC_50_ of 101.23, 68.83, 4.88, 3.24 μg/mL and against H1N1 with IC_50_ of 51.29, 27.92, 4.24, and 3.06 μg/mL respectively, whereas the IC_50_ value of the standard acyclovir against HVS-2 was 83.19 μg/mL and IC_50_ value of the standard ribavirin against H1N1 was 52.40 μg/mL .The methanol extracts of the roots (**Ext-3** and **Ext-5**) of both plants were characterized using UPLC/MS. A total of 73 metabolites were annotated, including fourteen diterpenoids, eleven flavonoids, ten phenolic acid conjugates, twelve fatty acids and their conjugates, five triterpenes and steroids, two sesquiterpenes, and six coumarins. The cytotoxicity and antiviral activities determined in the present work are explained by the existence of flavonoids, coumarins and diterpenes with commonly known cytotoxicity and antiviral activities.

## Introduction

One of the biggest plant families in the world is the Euphorbiaceae family, encompassing around 300 genera, 8000 species, and five subfamilies across the globe. These genera are primarily found in tropical and subtropical regions and are recognized as a significant source of poisons and medications^1,2^. In Yemen, there are 17 genera in the Euphorbiaceae family. (*Andrachne* L., *Cephalocroton* Del., *Flueggea* Willd, *Phyllanthus* L., *Clutia* L., *Chrozophora* Del., *Ricinus* L., *Meineckia* L., *Erythrococca* L., *Micrococca* Benth., *Acalypha* L., *Tragia* L., *Dalechampia* L., *Jatropha* L., *Bridelia* Muell-Arg ., *Croton* L. and *Euphorbia* L^3^.

*Jatropha* is a member of the Euphorbiaceae family and contains approximately 175 species, which are commonly used in traditional medicine in Africa, Asia, and Latin America to treat a variety of clinical ailments. *Jatropha* species are known to be a significant source of secondary metabolites with a wide range of biological processes^4,5^. In reality, the term "*Jatropha*" comes from the Greek language "jatros," which implies "doctor," and "trophe," which could be related to its widespread therapeutic usage^5^. Various species in this genus, including *Jatropha gossypiifolia, Jatropha mollissima, Jatropha curcas,* and *Jatropha elliptica,* have been studied for their medicinal uses, chemical constituents, and biological activities^5–9^. *Jatropha* species have been used in traditional medicine in many parts of Yemen to treat respiratory tract infections (RTI), as an antiseptic of wounds, as a hemostatic to stop bleeding, and for many other purposes^10^.

*Jatropha spinosa* and *J. variegata* are two mountainous plants that are indigenous to Yemen, *J. variegata* is native in Yemen, while *Jatropha spinosa* is indigenous to Yemen and other regions in Saudi Arabia, Somalia, and Djibouti. *Jatropha* species are represented in Yemen by seven species which are *J. curcus* L*, J. glauca* Vahl*., J. spinosa* (Forssk.)Vahl*, J. pelargoniifolia* Courb*., J. unicostata* Balf*., J. dhofarica* R.Sm and *J. variegata (Forssk.)*Vahl*.*^3^.

However, there is no adequate chemical and biological research on these two species. Nonetheless, there is a significant amount of research on other *Jatropha* species. *J. variegata* (Forsk.) Vahl has traditionally been utilized for antimicrobial and hemostatic actions in Yemeni folk medicine, as well as wound management^11^. Previous research has shown that *J. variegata* has antibacterial and antioxidant properties, most likely because of the existence of bioactive steroids and flavonoids^12^. Five flavonoids, namely kaempferol 3-*O*-L-rhamnopyranoside, 3-*O*-L-arabinopyranoside, 3-*O*-*β*-D-glucopyranoside, 3-*O*-*β*-D-galactopyranoside, and kaempferol, had previously been isolated from the stem of *J. variegata*^13^. Kaempferol and its glycosides have been demonstrated to have antioxidant, anti-inflammatory, anticancer, antibacterial, antimicrobial, neuroprotective, and hepatoprotective activities^14^, implying that flavonoids may contribute to the plant's medicinal potential.

Previous investigations of biological activities revealed that *J. variegata* exhibited high antibacterial, antioxidant, antifertility, and wound healing activities^15–17^. Meanwhile, *J. spinosa* has potent antimicrobial activity^18^. *Jatropha* plants have been widely studied in vitro to assess their cytotoxic effect rate against human cancer cell lines such as *J. gossypifolia*^19^, *J. curcas*^20^, *J. ribifolia*^21^, *J. multifida*^22^, and *J. podagrica*^23^. Furthermore, some *Jatropha* species, such as *J. curcas, J. gossypiifolia*, *J*. *multifida, J. unicostata*, and *J. podagrica* were found to have antiviral properties^24–28^.

In this research, the metabolites of both *J. spinosa* and *J. variegata* species were explored using ultra high-performance liquid chromatography-mass spectrometry to highlight similarities and differences in the chemical profile of both species. In light of these differences, the cytotoxicity and antiviral effects of both species were comparatively evaluated using numbers of cell lines.

## Materials and methods

### Chemicals

El Gomhouria for Drugs Co. (Cairo, Egypt) supplied the analytical-grade solvents required for the extraction and chromatographic separation processes. Silica gel F254 and RP silica gel were purchased from Sigma-Aldrich chemicals (Darmstadt, Germany), along with precoated TLC plates (20x20 cm). Doxorubicin and sulforhodamine-B (SRB) were bought from Sigma Chemical Co. in St. Louis, Missouri, in the United States. Methanol, formic acid, and acetonitrile were purchased from Fisher Scientific in Waltham, Massachusetts, USA. A Milli-Q system (Millipore, Bedford, MA, USA) was used to purify the water.

### Plant material

*Jatropha variegata* (leaves, stems, and roots) and *J. spinosa* (aerial parts and roots) were collected in January and February 2020 from Taiz government, Yemen and the collection procedure was in compliance with the national and international guidelines and legislation. The plants were identified by Agricultural Engineer Abd Alhabib Al-ghadasi in the Agriculture Research Institute of Yemen. Voucher specimens were maintained in the herbarium of Cairo University's Faculty of Pharmacy (PN-2–11-2022Ì and 2–11-2022ÌÌ).

### Extraction and fractionation of the crude extract

The air- dried powdered (1.5 kg) of *J. variegata* roots and (1.5 kg) of *J. spinosa* roots were exhaustively extracted with MeOH (3 × 3 L) using homogenizer (3 times each), and another amount (50g) of both of the leaves and stems of *J. variegata* and (50g) of stems of *J. spinosa* were extracted with MeOH (3X 300 mL) by cold maceration. The extracts were filtered and concentrated using a rotary evaporator at reduced pressure and at a temperature not exceeding 60°C. The crude extracts were stored in a refrigerator at -4°C until use. The process yielded MeOH extract of *J. variegata* leaves (**Ex-1**, 1.8g; 3.6%), MeOH extract of *J. variegata* stems (**Ext-2**, 2g; 4%) , MeOH extract of *J. variegata* roots (**Ext-3, **110g; 73%), MeOH extract of *J. spinosa* stems (**Ext-4**, 3.6g; 7.2%) and MeOH extract of *J. spinosa* roots (**Ext-5,** 125g; 8.3%). Both methanol extracts (**Ext-3 and Ext-5**) were suspended in H_2_O (900 mL) and extracted with CH_2_Cl_2_ (3 × 600 mL). The solvent was removed by distillation to yield CH_2_Cl_2_ fraction of *J. variegata* roots (**Fr-6,** 21 g; 1.4%) and to yield CH_2_Cl_2_ fraction of *J. spinosa* roots (**Fr-7,** 19 g; 1.2%)*.* The aqueous remaining fractions of *J. variegata* (49g; 3.2%), and *J. spinosa* (65g; 4.3%) were passed through Diaion-HP20 (4 × 20 cm) and eluted with 100% water (500 mL), 50% MeOH (500 mL) followed with 100% MeOH (750 mL) to give **Fr-8** (15g), **Fr-9** (26g), **Fr-10** (4g) and **Fr-11**(4g).

The methanolic extracts(s) were analyzed by Ultra High-Performance Liquid chromatography/diode array detector/Quadrupole Time-Of-Flight Mass Spectrometry (UHPLC/DAD/QTOF/MS) and were biologically tested.

### LC–MS analysis

#### UHPLC-QTOF-MS/MS profiling of the crude extracts and fractions

Using an Agilent UHPLC-MS system made up of an Agilent 1290 Infinity II UHPLC connected to an Agilent 6545 ESI-Q-TOF–MS in both negative and positive modes, aliquots (1 µl) of methanol extracts at a concentration of 2 mg/mL were examined. The following were the chromatographic conditions: Flow rate is 0.4 mL/min, a Kinetex phenyl-hexyl (1.7 µm, 2.1 50 mm) column was eluted with an isocratic elution of 90% H_2_O/MeCN for 1 min, followed by a 6-min linear gradient elution to 5% H_2_O/MeCN with 0.1% isocratic formic acid as modifier. The capillary temperature was set to 320 °C, the source voltage to 3.5 kV, and the sheath gas flow rate was set to 11 L/min for ESI. Ions identified with an intensity greater than 1000 counts per scan at 6 scans/s, with an isolation width of 1.3 m/z, a maximum of 9 selected precursors per cycle, and ramping collision energy (5 m/z/100 + 10 eV). Purine C_5_H_4_N_4_ [M + H]^+^ ion (*m/z* 121.0508) and hexakis (1H,1H,3H-tetrafluoropropoxy)-phosphazene C_18_H_18_F_24_N_3_O_6_P_3_ [M + H]^+^ ion (*m/z* 922.0098) were used as internal lock masses for positive mode while TFA C_2_HF_3_O^2^ [M − H]^−^ ion (*m/z* 112.9855) and hexakis (1H,1H,3H-tetrafluoropropoxy)-phosphazene C_18_H_18_F_24_N_3_O_6_P_3_ [M + TFA − H]^−^ion (*m/z* 1033.9881) were used as internal lock masses for negative mode.

### Biological activity

#### Cell culture

Nawah Scientific Inc. (Mokatam, Cairo, Egypt) provided the hepatocellular carcinoma (HepG2), lung cancer (A-549) , breast adenocarcinoma (MCF-7) cells and human skin fibroblast (HSF) cells for this study. The cells were kept in DMEM medium supplemented with 100 mg/mL streptomycin, 100 units/mL penicillin, and 10% heat-inactivated fetal bovine serum at 37 °C in a humidified, 5% (v/v) CO_2_ atmosphere. Additionally, cells for HSV type 2, H1N1, and Vero were purchased from Nawah Scientific Inc. in Egypt. In DMEM medium supplemented with 10% fetal bovine serum and 0.1% antibiotic/antimycotic solution, Vero cells were cultured. We tested the cytotoxicity prior to this assay by seeding cells at a density of 2 × 10^4^ cells/well in a 96-well culture plate. The following day, we cultured the cells in culture media containing serially diluted samples for 48 h before removing the medium and washing the cells with PBS.

### Cytotoxicity assay

The SRB assay was used to determine cell viability as described by Skehan^29^. In 96-well plates, aliquots of 100 L of cell suspension (5 × 103 cells) were seeded and incubated in complete media for 24 h. The cells were subsequently treated with 100 L medium containing various doses of medicines. Cells were fixed 72 h after drug exposure by replacing medium with 150 L of 10% TCA and incubating at 4 °C for 1 h. After removing the TCA solution, the cells were washed 5 times with distilled water. Aliquots of 70 L SRB solution (0.4% w/v) were added and incubated at room temperature for 10 min in the dark. The plates were cleaned three times with 1% acetic acid and air-dried overnight.

### Antiviral assay

Cell viability was determined by inhibiting the Cytopathic Effect (CPE) with crystal violet. Vero E6 cells and Vero cells were cultured in DMEM medium with 10% fetal bovine serum and 0.1% antibiotic/antimycotic solution. Gibco BR (Grand Island, NY, USA) provided the antibiotic and antimycotic solution, trypsine-EDTA, fetal bovine serum, and DMEM medium. Schmidtke et al. described the crystal violet method for evaluating antiviral and cytotoxicity activity^30^. In brief, one day before infection, Vero cells were planted at a density of 2 × 104 cells/well in a 96-well culture plate. The culture media was withdrawn the next day, and the cells were rinsed with PBS. The crystal violet method was used to measure H1N1 infectivity, which monitored CPE and allowed the percentage of cell viability to be estimated. The antiviral potency of each test sample was assessed using a concentration range of 0.1–100 g/mL that had been twice diluted. The assay was conducted with viral controls (virus-infected, non-drug treated cells) and cell controls (non-infected, non-drug treated cells). The culture plates were cultured for three days at 37 °C in 5% CO2, and the progression of the cytopathic effect was seen under a light microscope. The cell monolayers were fixed and stained using a 0.03% crystal violet solution in 2% ethanol and 3% formalin after being cleaned with PBS. After the plates had dried and been washed, a spectrophotometric measurement at 540/630 nm was used to determine the optical density of each individual well. This allowed for the calculation of the percentage of antiviral activities of the tests compounds according to Pauwels, et al.^31^ using the following equation:

Antiviral activity = [(mean optical density of cell controls − mean optical density of virus controls)/ (optical density of test − mean optical density of virus controls)] × 100% using the DIAS. Based on these results, the 50% CPE inhibitory dose (ID_50_) was calculated.

## Results and discussion

### UHPLC-DAD-QTOF-MS/MS profiling of methanol extracts

The metabolite profiling of the methanol extracts of roots of *J. variegata* and *J. spinosa* (**Ext-3 and Ext-5***,* respectively) with potential cytotoxicity and antiviral activities were analyzed using UHPLC-DAD-QTOF-MS/MS analysis. Some classes of compounds, such as sesquiterpenes and diterpenes, were well-identified in the positive ionization mode, whereas flavonoids, phenolic acids, and quinic acid derivatives could be detected in the negative ionization mode^32^.

**Figure 1:**
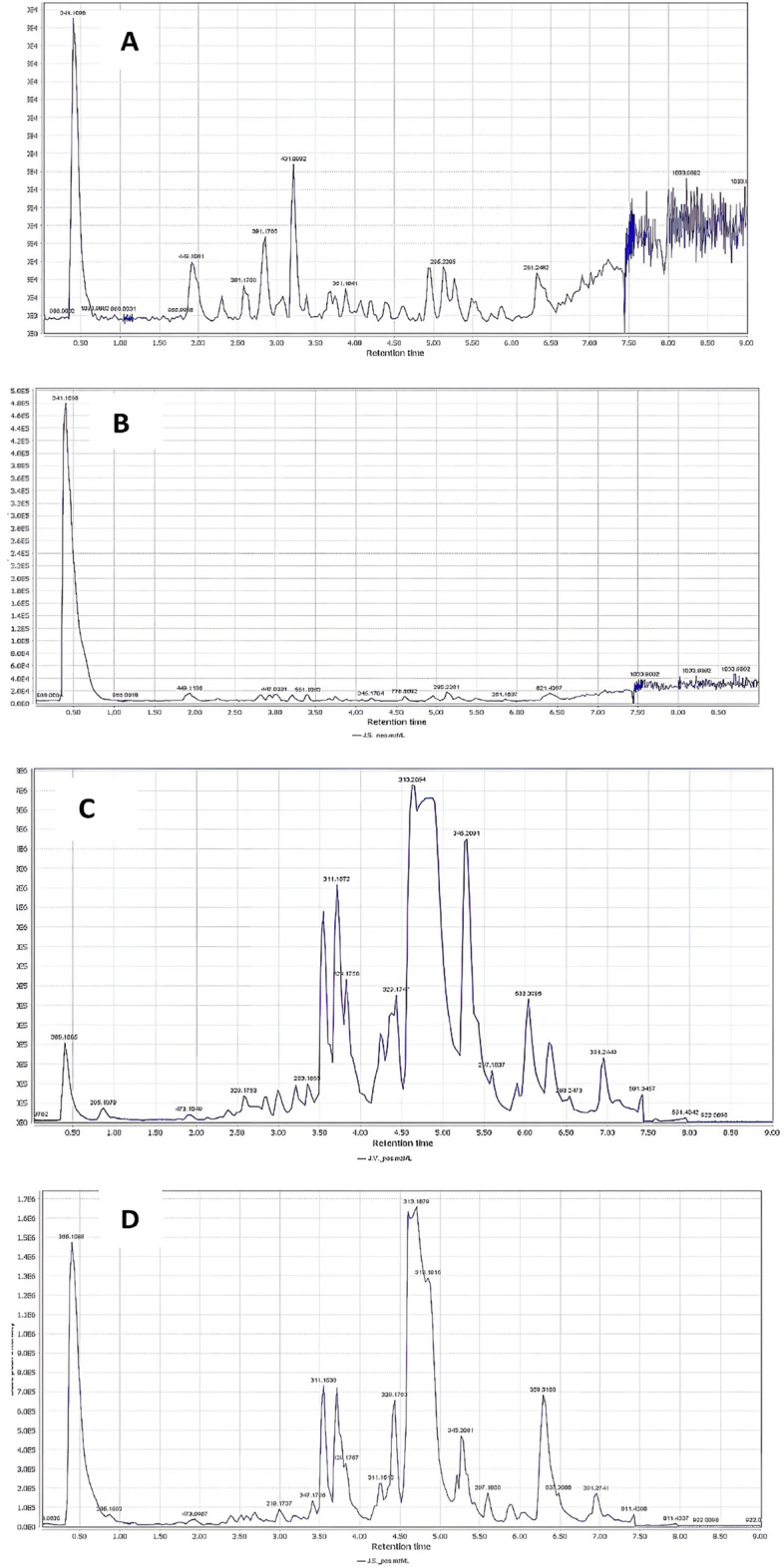
Base peak chromatograms (BPC) of the MeOH extracts of *J. variegata* and *J. spinosa* roots in negative (**A**, **B**) and positive (**C**, **D**) ionization modes, respectively.

Figure 1 depicted the base peak chromatograms (BPC) of both species' MeOH extracts in negative and positive modes. In both extracts, the analytical setup in both ESI modes resulted in the detection of 73 metabolites (Table 1). The UV spectra, accurate mass, and MS fragmentation patterns were used to characterize the metabolites. The mass spectra were also compared to those in the phytochemical dictionary of natural products database (CRC) and the published literature^33–44^. The identified compounds are represented by 10 phenolic acids and phenolic derivatives, 12 fatty acids, 11 flavonoids, 6 coumarins, 14 diterpenes, 7 nitrogenous compounds, 5 triterpenes and steroids, 2 sesquiterpenes and 6 miscellaneous compounds.

**Table 1:** Metabolites identified in the methanol extracts of *J. variegata ***(**JV**)** and *J. spinosa*** (**JS**)** in positive ionization mode [M + H]^+^ and negative ionization mode [M-H]^-^.

No	Rt (min)	Compound name	JV	JS	Molecular formula	Error (ppm)	[M + H]^+^	Fragment ions	[M-H]^−^	Fragment ions
**Flavonoids**										
1	0.88	Eriodictyol-*O*-hexoside	**√**	**√**	C_21_H_22_O_11_	0.25			449.1084	287, 269, 259, 178.99, 155, 125
2	1.88	Eriodicytol	**√**	**√**	C_15_H_12_O_6_	− 1.6	289.0693	271, 253, 243, 229, 215, 149, 107	–	–
3	2.44	Homoorientin		**√**	C_21_H_20_O_11_	− 1.67			447.0924	357, 327, 298, 285, 269
4	2.80	Isoquercitrin Quercetin-*O*- hexoside)		**√**	C_21_H_20_O_12_	− 0.92	–	–	463.0873	301, 300, 271, 255, 179, 151
5	2.83	Taxifolin		**√**	C_15_H_12_O_7_	2.37	–	–	303.0508	285, 259, 217, 179, 177, 153, 137, 125
6	2.97	Quercitrin		**√**	C_21_H_20_O_11_	− 1.59	–	–	447.0924	301, 300, 271, 255, 179, 151
7	3.02	Kaempferol	**√**	**√**	C_15_H_10_O_6_	0.58	287.0540	_258, 241, 231, 227, 213, 185, 165, 153, 121, 97, 77_	–	–
8	3.03	Quercetin	**√**	**√**	C_15_H_10_O_7_	− 2.2	303.0489	285, 275, 257, 247, 229, 201,165	–	–
9	3.10	Naringenin-*O*- hexoside	**√**		C_21_H_22_O_10_	− 1.5	–	–	433.1142	287, 269, 259, 180, 152, 151, 125, 107
10	3.13	Vitexin	**√**		C_21_H_20_O_10_	− 1.5	–	–	431.0983	341, 311, 284, 283, 269, 255
11	3.9	Naringenin		**√**	C_15_H_12_O_5_	− 1.55			271.0600	204, 151, 119, 107
**Phenolic acid conjugates**										
12	0.48	*β*-Glucogallin 1-*O*-galloyl- hexoside		**√**	C_13_H_16_O_10_	− 2.76	–	–	331.0670	211, 169, 151, 125, 123
13	0.51	Quinic acid	**√**	**√**	C_7_H_12_O_6_	0.61	–	–	191.0556	173, 93, 85
14	0.76	Catechol	**√**	**√**	C_6_H_6_O_2_	0.38	–	–	109.02888	108, 91
15	0.85	Protocatechuic acid	**√**	**√**	C_7_H_6_O_4_	− 1.35	–	–	153.0181	133, 109, 96
16	1.2	*p*-Hydroxybenzoic acid	**√**	**√**	C_7_H_6_O_3_	3.51	–	–	137.0241	193
17	1.58	Benzoic acid	**√**	**√**	C_7_H_6_O_2_	4.8	–	–	121.0289	121 ,93, 92
18	1.88	Corilagin/-*O*-Galloyl-6-*O*-luteoyl-D- hexoside		**√**	C_27_H_22_O_18_	− 0.76	633.0722	463, 415, 300, 275	–	–
19	1.94	Octyl benzoate	**√**		C_15_H_22_O_2_	0.06	235.1681	217, 179, 123, 121, 107, 69, 57	–	–
20	2.44	*p*-Coumaroyl-caffeoylglycerol		**√**	C_21_H_20_O_8_	1.39	401.1557	401, 383, 355, 270, 249, 199, 152	–	–
21	6.95	Tetradecylferulate	**√**	**√**	C_24_H_38_O_4_	0.51	391.2832	167, 149		
**Coumarins**										
22	2.08	Fraxetin	**√**	**√**	C_10_H_8_O_5_	− 0.57	209.0437	194, 181, 163, 149, 135, 121	–	–
23	2.85	Fraxidin	**√**	**√**	C_11_H_10_O_5_	0.44	223.0595	208, 190, 162, 150, 134	–	–
24	3.60	Coumarin	**√**	**√**	C_9_H_6_O_2_	4.72	147.1161	132, 119, 105, 103, 91	–	–
25	4.18	3,4-dihyro Coumarin	**√**	**√**	C_9_H_8_O_2_	− 0.028	149.0950	133, 121, 119, 107, 105, 93, 91, 77	–	–
26	4.28	Propacin	**√**	**√**	C_20_H_18_O_7_	3.36	371.1113	209, 207, 194, 176, 164, 149, 131, 103	–	–
27	4.52	7-Mehylcoumarine	**√**	**√**	C_10_H_8_O_2_	0.03	161.0589	146, 133, 121, 119, 105, 103, 91	–	–
**Nitrogenous compounds**										
28	0.38	Arginine	**√**		C_6_H_14_N_4_O_2_	2.05	175.1181	158, 131, 116, 70	–	–
29	0.40	N-Glucosylarginine	**√**		C_12_H_24_N_4_O_7_	0.82	337.1704	319, 242, 216, 198, 175, 158, 130, 114, 70	–	–
30	0.43	Choline hexoside	**√**	**√**	C_11_H_23_NO_6_	11.3	266.1223	230, 212, 128, 116, 104, 98, 87	–	–
31	0.44	Betaine	**√**	**√**	C_5_H_11_NO_2_	0.11	118.0855	118, 59	-	–
32	0.45	Indol carboxyaldehyde	**√**		C_9_H_7_NO	2.11	146.0914	128, 111, 104, 86, 72, 60	-	–
33	0.47	Choline	**√**	**√**	C_5_H_13_NO	− 0.08	104.1064	60, 59, 58, 54	–	–
34	0.78	Adenine	**√**		C_5_H_5_N_5_	− 4.27	136.1114	136, 120, 119	–	–
**Diterpenes**										
35	2.80	4,12-deoxyphorbol	**√**	**√**	C_20_H_24_O_4_	− 1.41	329.1735	311, 301, 293, 287, 283, 269 265, 163	–	–
36	3.27	Dihydroxy-13- methylpodocarpane-triene	**√**	**√**	C_18_H_26_O_2_	1.22	275.1609	257, 239, 201, 173, 157, 161, 119	–	–
37	3.49	4,9,12-trideoxyphorbol	**√**	**√**	C_20_H_22_O_3_	− 1.39	311.1631	293, 283, 265, 251, 227, 187 159, 125	–	–
38	3.52	12-deoxyphorbol	**√**	**√**	C_20_H_26_O_5_	− 1.47	347.1839	329, 311, 301 , 293, 287, 283, 269, 163	–	–
39	4.32	Curcusone C or D	**√**	**√**	C_20_H_24_O_3_	− 2.84	313.1927	298, 271, 257, 229, 203,173, 159, 119, 81		
40	4.46	Jatrocurcasenone E	**√**	**√**	C_20_H_26_O_4_	- 2.66	331.1891	313, 285, 241 , 213, 189, 163, 147, 125	–	–
41	4.54	Jatrophone	**√**	**√**	C_20_H_24_O_3_	2.01	313.1788	295, 285, 257, 239, 211, 189, 175, 161, 147, 125	–	–
42	4.74	2-Epimacroripremyrisinoe A	**√**	**√**	C_20_H_26_O_4_	0.93	331.1890	313, 285, 267, , 243, 211, 163, 121	–	–
43	5.13	Spruceanol	**√**	**√**	C_20_H_28_O_2_	0.16	301.2149	284, 260, 243, 227, 213, 218, 199, 185, 177 , 149, 133, 121, 93	–	–
44	5.21	Jatropholone B	**√**	**√**	C_20_H_24_O_2_	0.87	297.1837	241, 229, 213, 199, 185, 173	–	–
45	5.57	Multifidone	**√**	**√**	C_20_H_28_O_3_	0.77	315.1940	297, 269, 255, 241, 231, 199, 145, 133, 123 81	–	–
46	6.23	Ferruginol	**√**	**√**	C_20_H_30_O	0.08	287.2356	269, 241, 215, 201, 189, 175, 163,147, 145, 121, 109, 69	–	–
47	6.45	Peditithin H	**√**	**√**	C_36_H_44_O_10_	− 1.25	637.3044	619, 581, 525, 469	–	–
48	6.54	Phorbol 12,13 diacetate	**√**	**√**	C_24_H_32_O_8_	10.2	449.3038	431, 414, 387, 346, 333, 313, 175, 121, 95,, 69		
**Triterpenoids and steroids**										
49	6.62	Dioxo-olean-12-ene	**√**	**√**	C_30_H_46_O_2_	2.05	439.3556	393, 301, 245, 203, 198, 147, 121, 109, 95, 81	–	–
50	6.76	Taraxasterol	**√**	**√**	C_30_H_50_O	7.1	427.3558	409, 381, 271, 253, 229, 211, 175, 157, 133, 83, 69	–	–
51	6.94	Stigmasterol	**√**	**√**	C_29_H_48_O	4.58	413.2649	395, 301, 213, 189, 159, 157, 135, 107, 83, 69	–	–
52	6.84	Stigma-4-en-6-beta-ol-3-one	**√**	**√**	C_29_H_48_O_2_	0.62	429.3710	411, 95	–	–
53	7.36	Stigmastane 3,6 dione	**√**	**√**	C_29_H_48_O_2_	1.67	429.3710	219, 165	–	–
**Fatty acid and their conjugates**										
54	0.87	Hydroxy-octadecatetrienoic acid	**√**	**√**	C_18_H_30_O_3_	3.67	295.1866	277, 259, 251, 233, 175	–	–
55	1.20	Oxo-phytodienoic acid	**√**	**√**	C_18_H_28_O_3_	0.4	293.1708	275, 257, 258, 247, 195	–	–
56	1.25	Oxo-octadecatetraenoic acid	**√**	**√**	C_18_H_26_O_3_	0.13	291.1553	273, 249, 207, 147	–	–
57	2.97	Octadecatetraenoic acid	**√**	**√**	C_18_H_28_O_2_	5.57	277.1761	259, 217, 188, 175, 149, 107	–	–
58	3.60	9,12,13-Trihydroxy-10,15-octadecadienoic acid	**√**	**√**	C_18_H_32_O_5_	4.06	–	–	327.2167	309, 211, 183, 171
59	3.71	Hydroxyl octadecanedioic acid	**√**	**√**	C_18_H_34_ O_5_	2.62	–	–	329.2324	314, 298, 285, 271, 211, 171
60	5.15	Linolenic acid	**√**	**√**	C_18_H_30_O_2_	− 0.65	279.2306	149, 123, 109, 95, 81, 67		
61	5.85	2-Monopalmitate monoglycerid 16:0	**√**	**√**	C_19_H_38_O_4_	00.02	331.2831	313, 257, 241, 239, 123, 109, 95, 57	–	–
62	6.08	Linoleic acid	**√**	**√**	C_18_H_32_O_2_	0.79	–	–	279.2325	279
63	6.22	Palmati c acid	**√**	**√**	C_16_H_32_O_2_	0.56	–	–	255.2321	255
64	6.23	Monosteirin	**√**	**√**	C_21_H_42_O_4_	1.91	359.3143	341, 310, 285, 230, 207,173, 137, 123, 109	–	–
65	6.33	Oleic acid	**√**	**√**	C_18_H_34_O_2_	3.51			281.2479	281
**Sesquiterpenoids**										
66	3.32	Cuparene	**√**	**√**	C_15_H_22_	1.4	203.1782	188, 175, 161, 147, 133, 119, 105, 69	–	–
67	4.38	Dihydro-pulicaric acid	**√**	**√**	C_15_H_22_O_3_	4.28	–	–	249.1492	205, 187
**Miscellaneouse compounds**										
68	0.35	Sucrose	**√**	**√**	C_12_H_22_O_11_	− 1.67	341.1086	179, 161, 119, 89	–	–
69	0.40	Ethyl aconitate dihexoside	**√**	**√**	C_20_H_30_O_16_	− 2.01	527.1563	365, 347, 203, 185	–	–
70	0.43	Ethyl aconitate hexoside	**√**	**√**	C_14_H_20_O_11_	2.06	365.1041	203, 185	–	–
71	0.86	Emodin	**√**	**√**	C_15_H_10_O_5_	− 5.1	271.0588	243, 225, 215, 197	–	–
72	2.74	Azelaic acid	**√**	**√**	C_9_H_16_O_4_	0.1	187.0974	125	–	–
73	3.05	Eudesmin/ Epieudesmin	**√**		C_22_H_26_O_6_	2.17	387.1755	309, 278, 255, 187, 171, 158, 147	–	–

### Phenolic compounds

The UHPLC-MS analysis of tested samples revealed the presence of 11 flavonoids, 10 phenolic acid conjugates, 6 coumarins, and 1 lignan were identified. Flavonoids were found primarily as C-linked hexoses (5 flavonoids), which were easily distinguished by a neutral loss of 120 Da., as observed in compounds as homoorientin (3) and vitexin (10), vitexin was differentiated from its structural isomer isovitexin by the intensity of the fragment ion at 283 *m/z*, also homoorientin was differentiated from orientin by the intensity of the fragments 298 and 327 *m/z*.

Flavonoids *O*-glycosides were characterized by the neutral loss of 162 Da for hexoses as observed in eriodictyol-hexoside **(1)** quercetin-*O*-hexoside **(4)** and naringenin *O*-hexoside **(9).** Most of these flavonoids were detected in *Jatropha* species^33,35,36^.

Phenolic acids in *J. variegata* and *J. spinosa* extracts were detected and eluted first in the chromatogram, as seen in Table 1. Among the phenolic acid esters that have been annotated, only tetradecyl ferulate has been documented in the genus and has been isolated from both *J. curcas*^45^, and *J. multifida*^46^, and was previously detected in *J. integerrima*^33^.

### Nitrogenous compounds

In *J. variegata* and *J. spinosa* extracts, seven simple nitrogenous compounds were identified, including choline and its glycoside. Other simple nitrogenous compounds including arginine and *N*-gucosylarginine, as well as indol carboxyaldehyde, betaien, and adenine, were also detected in the extract.

### Terpenoids

The most frequent secondary metabolite group found in *J. variegata and J. spinosa* roots was composed of terpenoids, including two sesquiterpenes, five triterpenes and steroids, and 14 metabolites of diterpenes. Free diterpenoids esters, predominantly phorbol esters, were the first to be eluted in this investigation at retention time (2.8–3.5 min), which could easily be distinguished because to the neutral loss of acetic acid (60 Da), as seen in 12-deoxyphorbol, 4,9,12-trideoxyphorbol, and 12-deoxyphorbol. Additionally, at retention time 4.3–6.2 min, free diterpenoids were eluted, and were tentatively identified as jatrophone, curcusone, spruceanol, mutifidone, jatropholone B, jatrocurcasenone E, and ferruginol, containing one to three oxygen atoms. Most of these diterpenes were isolated and previously identified in the genus *Jatropha*^36,37^.

Ferruginol diterpene which was the first compound identified in the genus *Jartopha* by observing a mass ion at *m/z* 287.2356 [M + H]^+^ and fragments at *m/z* 201 [M + H-C_6_H_14_ ]^+^, 189 [M + H-C_7_H_14_] ^+^, 175 [M + H-C_8_H_16_]^+^. This chemical has previously been discovered in Euphorbiaceae plants and in *Juniperus procera* leaves and stems^47^.

Five triterpenes and steroids have been annotated in the UHPLC/MS chromatograms of *J. variegata* and *J. spinosa* including stigmasterol (**51**), stigmastane 3,6 dione (**53**), taraxasterol(**50**), dioxo-olean-12-ene (**49**) and stigmast-4-en-6- β-ol-3-one (**52**), all of which were earlier isolated and identified in the genus *Jatropha*^4^.

Two sesquiterpenes were identified in both species, namely cuparene (**66**) and dihydro-pulicaric acid (**67**). Dihydro-pulicaric acid (**67**), a dihydroderivative of pulicaric acid, was identified in the genus *Jatropha* and in the family Euphorbiaceae for the first time, but was previously identified in *Pulicaria crispa* and *P. incise*^48^ . It was identified by observing mass ion at *m/z* 249.1492 [M-H]^-^ and fragments at *m/z* 205 [M-H-CO_2_ ]^-^, 187 [M-H-CO_2_-H_2_O). Cuparene sesquiterpene was not identified from genus *Jatropha* but was identified in *Euphorbia macrorrhiza*^49^, according to the fragmentation pattern with mass ions at *m/z* 203.1782 [M + H]^+^, 188 [M + H-CH_3_]^+^, 175 [M + H- C_2_H_4_]^+^, 161 [M + H-C_3_H_6_]^+^, 147 [M + H- C_4_H_8_]^+^, and 133 [M + H- C_5_H_10_]^+.^.

### Fatty acids and their conjugates

Fatty acids were found in the form of free fatty acids and fatty acid glycerides, which accounted for 12 metabolites. The most prevalent fatty acids were unsaturated (C-18), including oxo-phytodienoic (C_18_H2_8_O_3_), linolenic (C_18_H_30_O_2_), oleic acid (C_18_H_34_O_2_), hydroxy-octadecatetrienoic acid (C_18_H_30_O_3_), oxo-octadecatetraenoic acid (C_18_H_26_O_3_), 9,12,13- trihydroxy-10,15-octadecadienoic acid (C_18_H_32_O_5_) and linoleic acid (C_18_H_32_O_2_). Only two fatty acids derivatives had annotations in the extracts including, 2-monopalmitate monoglyceride (16:0) and monosteirin. One saturated fatty acid was identified as palmitic acid (C_16_H_32_O_2_).

Five hydroxyfatty acids [hydroxy-octadecatetrienoic acid (**54**, C_18_H_30_O_3_), oxo-phytodienoic acid (**55,** C_18_H_28_O_3_), oxo-octadecatetraenoic acid (**56**, C_18_H_26_O_3_), 9, 12, 13-trihydroxy-10,15-octadecadienoic acid (**58**, C_18_H_32_O_5_), hydroxyl octadecanedioic acid (**59**, C_18_H_34_O_5_) were identified from the loss of two water molecules (2 × 18 amu). Based on the fragmentation pattern of hydroxy-octadecatetrienoic acid with ions at *m/z* 295.1866 [M + H]^+^, 277 [M + H-H_2_O]^+^, 259 [M + H- 2H_2_O]^+^, also the fragmentation pattern of oxo-phytodienoic acid with ions at *m/z* 293.1708 [M + H]^+^, 275 [M + H-H_2_O]^+^, 257 [M + H- 2H_2_O]^+^.

### Miscellaneous compounds

Six miscellaneous compounds were annotated in *J. variegata* and *J. spinoa* extracts including a sugar, a lignan, an anthraquinone, a dicarboxylic acid and two tricarboxylic acid derivatives, namely sucrose, eudesmin/epieudesmin, emodine, azalic acid, ethyl aconitate hexoside, and ethyl aconitate dihexoside, respectively. Most of these compounds were identified in the genus *Jatropha*^33,35^. Only emodin anthraquinone was not detected in the genus *Jatropha* but was identified in Euphorbiaceae family^50^. The elimination of CO to create m/z 243 was followed by the loss of one hydroxyl group to produce m/z 225 in the fragmentation of emodin (C_15_H_10_O_5_).

### In vitro cytotoxic activity

**Figure 2:**
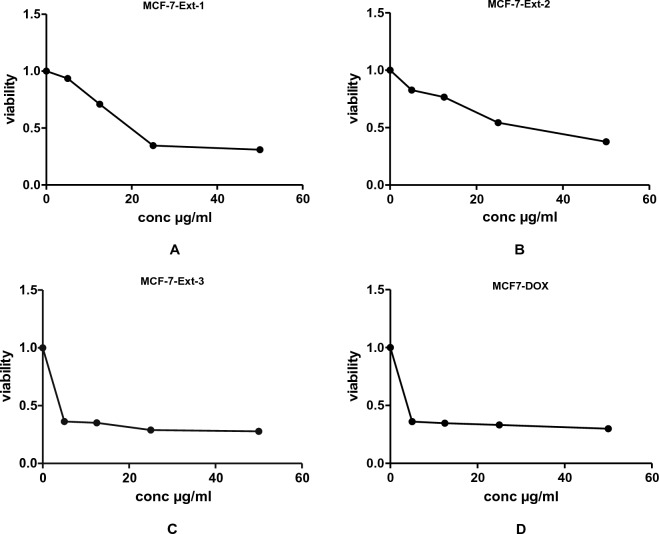
The effects of methanol extracts (**A**—leaves: Ext-1, **B**—stem: Ext-2 and **C**—roots: Ext-3) of *J. variegata* on the cytotoxicity in MCF- 7 cell line.

**Figure 3:**
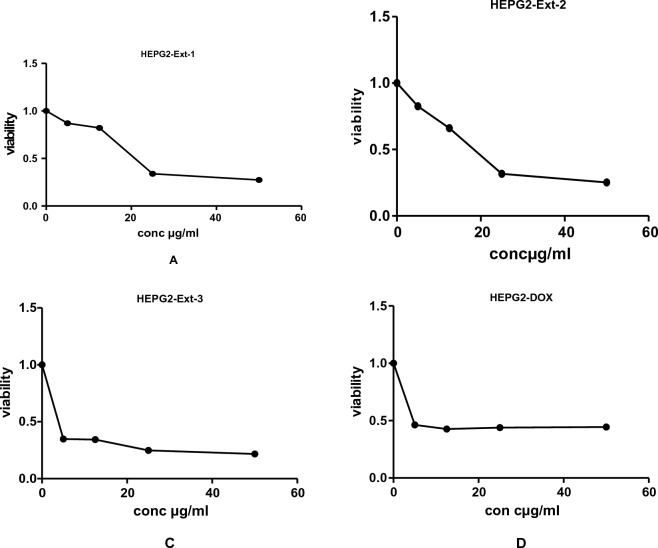
The effects of methanol extracts (**A**—leaves: **B**—Ext-1, **C**—stem: Ext-2 and **D**—roots: Ext-3) of *J. variegata* on the cytotoxicity in HEPG-2 cell line.

**Figure 4:**
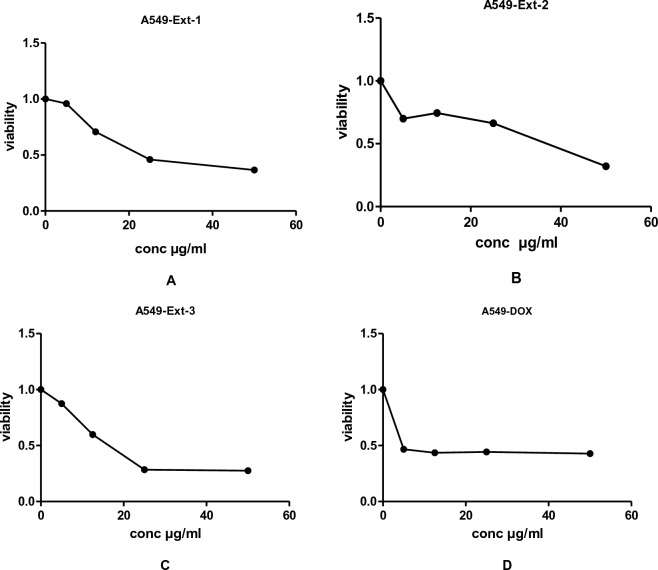
The effects of methanol extracts (**A**—leaves: Ext-1, **B**—stems: Ext-2 and **C**—roots: Ext-3) of *J. variegata* on the cytotoxicity in A549 cell line.

**Figure 5:**
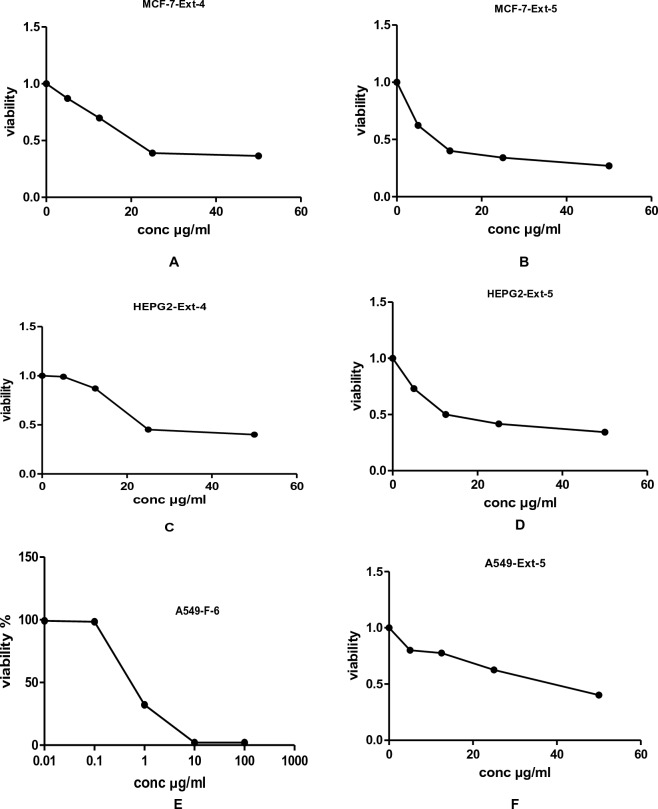
The effect of methanol extracts (**A**, **C**, **E**—stems: Ext-4 and **B**, **D**, **F**—roots: Ext-5) of *J. spinose* on the cytotoxicity in MCF-7, HEPG2 and A549 cell lines respectively.

**Figure 6:**
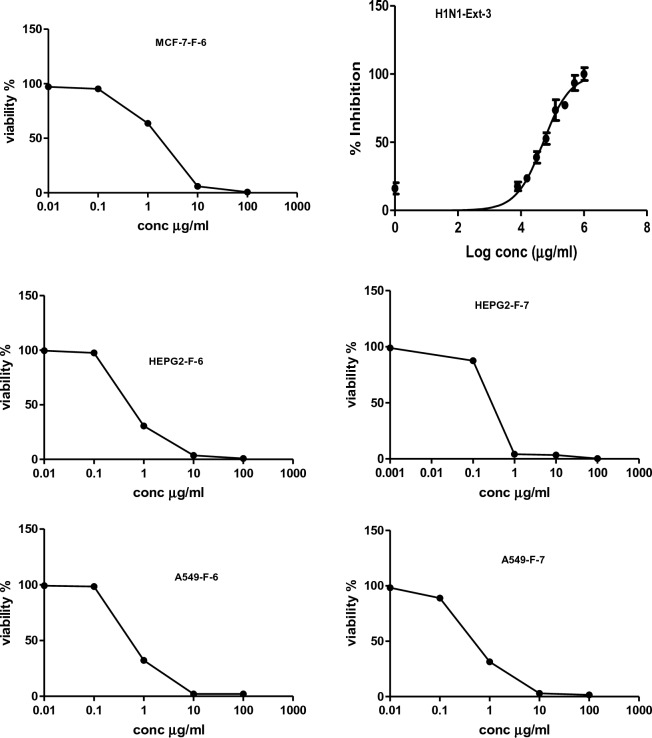
The effect of CH_2_CI_2_ fractions of *J. variegata (F-6) and J. spinosa* (F-7) on the cytotoxicity in MCF-7, HEPG-2 and A549 cell lines.

This is the first report evaluating the in vitro cytotoxic activities of *J. variegata and J. spinosa* extracts against three human cancer cell lines, namely breast adenocarcinoma (MCF-7), hepatocellular carcinoma (HepG2), and lung cancer (A-549). The curves of the concentration response for the doxorubicin control drug, methanol extract and methylene chloride fractions against the three human cells lines are shown in Figs. 2, 3, 4, 5, 6. The results of the in vitro antiproliferative assay with human cancer cell lines are displayed in the Table 2.

**Table 2:** Cytotoxic activity of *J. variegata* (JV) and *J. spinosa* (JS) various MeOH extracts (Ext-1–5) and CH_2_Cl_2_ fraction (F-6 & F-7) against MCF-7, HEPG2, and A-549 cell lines.

Fraction/cell line	Human skin fibroblast (HSF) IC_50_(µg/mL)	Breast adenocarcinoma (MCF-7) IC_50_ (µg/mL)	SI HSF/IC50	Hepatocellular carcinoma (HepG2) IC_50_ (µg/mL)	SI HSF/IC50	Lung cancer (A-549) IC_50_ (µg/mL)	SI HSF/IC50
Ext-1(MeOH of leaves of *J. variegata*)	ND	20 ± 0.3223	ND	21 ± 0.03309	ND	23 ± 0.2858	ND
Ext-2 (MeOH of stems of *J. variegata*)	ND	32 ± 0.2447	ND	19 ± 0.3320	ND	37 ± 0.2429	ND
Ext-3 (MeOH of roots of *J. variegata*)	1.54 ± 0.07	4.0 ± 0.3060	0.39	4.0 ± 0.03230	0.385	16.5 ± 0.3313	0.093
Ext-4 (MeOH of aerial parts of *J. spinosa*)	ND	21 ± 0.2838	ND	24 ± 0.2941	ND	20 ± 0.2407	ND
Ext-5 (MeOH of roots of *J. spinosa*)	1.08 ± 0.02	10 ± 0.2960	0.11	12.5 ± 0.2676	0.086	38 ± 0.2220	0.028
F-6 (CH_2_Cl_2_ of roots of *J. variegata*)	0.69 ± 0.02	1.4 ± 0.0728	0.49	0.64 ± 0.0189	1.078	0.70 ± 0.037	0.986
F-7 (CH_2_Cl_2_ of roots of *J. spinosa*)	0.34 ± 0.01	1.0 ± 0.0310	0.34	0.24 ± 0.0038	1.417	0.53 ± 0.015	0.642
Doxorubicin	ND	3.83 ± 0.2984	ND	4.73 ± 0.249	ND	4.57 ± 0.2493	ND

Data is represented as mean ± standard deviation; SI: Selectivity index.

The potential cytotoxicity of eleven extracts and fractions of *J. variegata* (leaves, stem, and root) and *J. spinosa* (aerial parts and roots) including total methanol (**Ext-1, Ext-2** , **Ext-3, Ext-4, and Ext-5** ), CH_2_Cl_2_ fractions of *J. variegata* and *J. spinosa* roots (**F-6** and **F-7**), and diaion column fractions of *J. variegata* and *J. spinosa* roots ( **F-8:** 50%: MeOH, **F-9**: 100% MeOH, and **F-10:** 50% MeOH, and **F-11**: 100% MeOH) were assessed using sulforhodamine B (SRB) assay against MCF-7, HepG2, and A-549.

*J. variegata* and *J. spinosa* extracts and fractions were tested in vitro against three cell lines (MCF-7, HepG2, and A-549) compared with doxorubicin, and the results are demonstrated in Tables 2, 3 and Figs. 2, 3, 4, 5, 6. Based on the IC_50_ values of all tested samples, the CH_2_Cl_2_ fractions **F-6** and **F-7** demonstrated most strong cytotoxic activity. **F-6** showed IC_50_ values of 1.4 µg/ml, 0.64 µg/mL and 0.70 µg/mL against MCF-7, HepG2, and A-549 cell lines, respectively, whereas IC_50_ values of **F-7** were 1.0, 0.24 and 0.5 µg/mL against MCF-7, HepG2, and A-549 cell lines, respectively, whereas IC_50_ of doxorubicin was 3.83, 4.73, and 4.57 µg/ml against MCF-7, HepG2, and A-549 cell lines, respectively. **Ext-3** and **Ext-5** demonstrated greater anticancer activity against MCF-7, HepG2, and A-549 cell line, (4.0 and 10.0 µg/ml; 4.0 and 12.5 µg/ml; 16.5 and 38 µg/ml IC_50_, respectively) compared with other extracts, whereas DOX showed IC_50_ of 3.83, 4.73, and 4.57 µg/ml of IC_50_ against MCF-7, HepG2, and A-549 cell line, respectively. In case of fractions **F-8, F-9,** and **F-10**, no activity was observed against the three cell lines. The activities of **Ext-1, Ext-2**, and **Ext-4** extracts showed efficient activities for the three cell lines tested when compared to DOX, with in vitro antiproliferative activities ranging from 19.0 to 38.0 μg/mL.

**Table 3:** Cytotoxic activity of 100%MeOH fractions and 50%MeOH fractions of *J. variegata* (JV) and *J. spinosa* (JS) against MCF-7, HEPG2, and A-549 cell lines.

Fractions	Conc (µg/mL)	Breast adenocarcinoma (MCF-7)	Hepatocellular carcinoma (HepG2)	Lung cancer (A-549)
		Viability (%)	Viability (%)	Viability (%)
F-8 (50% MeOH of diaion fraction of JV)	10	98.52	97.84	98.42
	100	15.66	4.09	34.62
F-9 (50% MeOH of diaion fraction of JS)	10	98.43	98.14	98.94
	100	93.99	83.83	88.74
F-10 (100% MeOH of diaion fraction of JV)	10	98.43	86.79	98.38
	100	59.33	8.44	55.91
F-11 (100% MeOH of diaion fraction of JS)	10	97.28	97.31	98.80
	100	95.57	91.95	91.60

The cytotoxic activity of the most effective anticancer extracts (Ext-3 and Ext-5) and fractions (F-6 and F-7) was evaluated against non-cancerous cells, human skin fibroblast (HSF) cell line to test their selectivity for cancer cells and results are shown in Table 2. The tested extracts and fractions showed considerable cytotoxic activity against HSF cells with IC_50_ values of 1.54 ± 0.07, 1.08 ± 0.02 and for Ext-3 and Ext-5 and 0.69 ± 0.02 and 0.34 ± 0.01 for **F-6** and **F-7**, respectively. Therefore, the selectivity index (SI) was calculated for the active extracts (Ext-3 and Ext- 5) and fractions (**F-6** and **F-7**) and shown in Table 2. The potent antiproliferative activity of **F-6** was accompanied with high selectivity in case of HepG2 and A-549 (SI 1.078 and 0.986, respectively) and a moderate selectivity for MCF-7 (SI 0.49). Also, **F-7** displayed strong SI in case of HepG2 (SI 1.417).

The diverse bioactive metabolites in *Jatropha* species may be responsible for the variations in the efficiency of the extracts against cancer cells. So, using UHPLC-DAD-QToF, untargeted metabolite profiling of the MeOH extracts was carried out. The LC–MS profile of the extracts showed the presence of six coumarins, including propacin, fraxidin, fraxetin, 3,4-dihydrocoumarine, coumarin, and 7-methyl coumarin, as well as major components including diterpenes (seen in positive mode) and flavonoids (indicated in negative ion mode). These coumarins, flavonoid and terpenoids enhance the active MeOH extracts as revealed in LC–MS and are well known for their anticancer properties.

### In vitro antiviral activity

**Figure 7:**
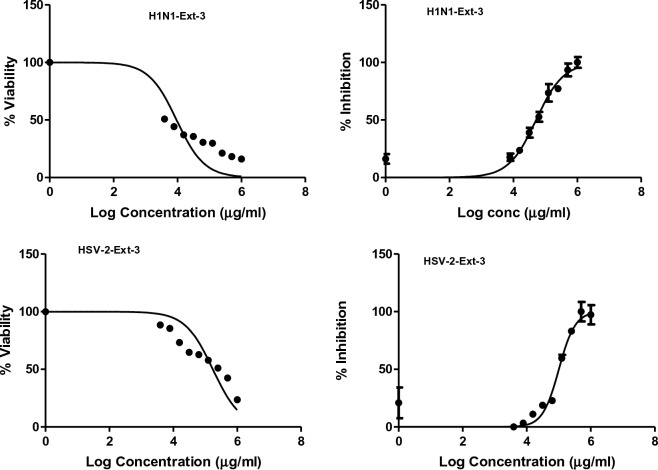
The effects of methanol extract of *J. variegata roots (Ext-3)* on the viral inhibition in H1N1 and HSV-2 cell lines.

**Figure 8:**
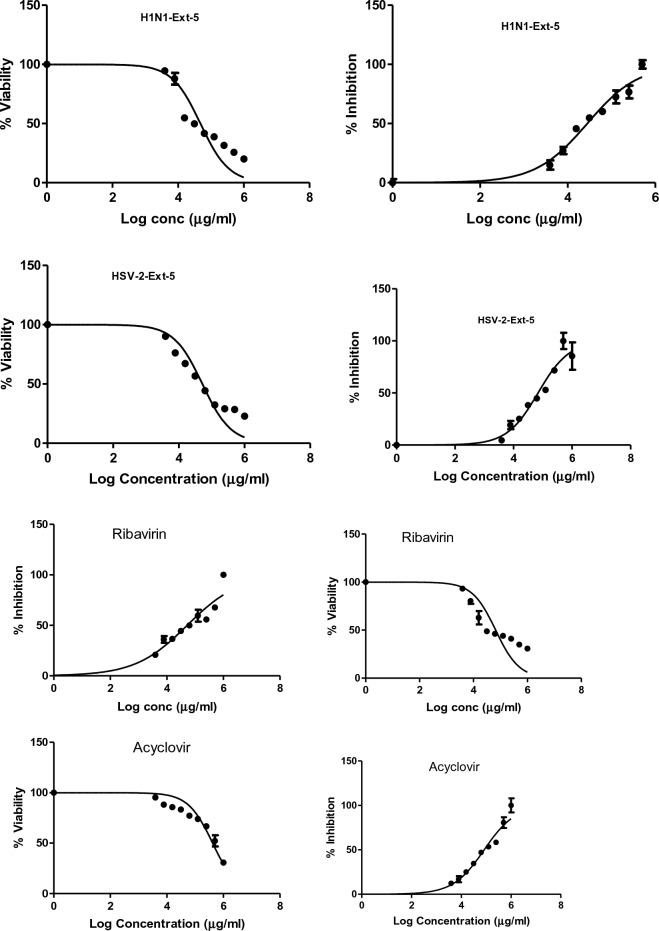
The effects of methanol extract of *J. spinosa roots* (Ext-5) on the viral inhibition in H1N1 and HSV-2 cell lines.

**Figure 9:**
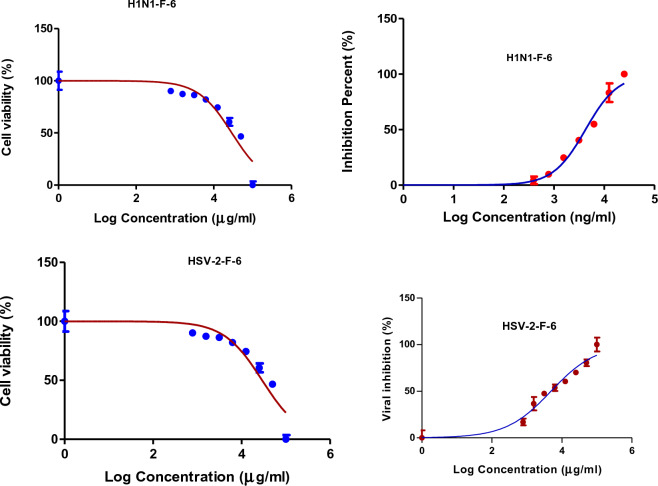
The effects of CH_2_CI_2_ fraction of *J. variegata roots* (F-6) on the viral inhibition in H1N1 and HSV-2 cell lines.

**Figure 10:**
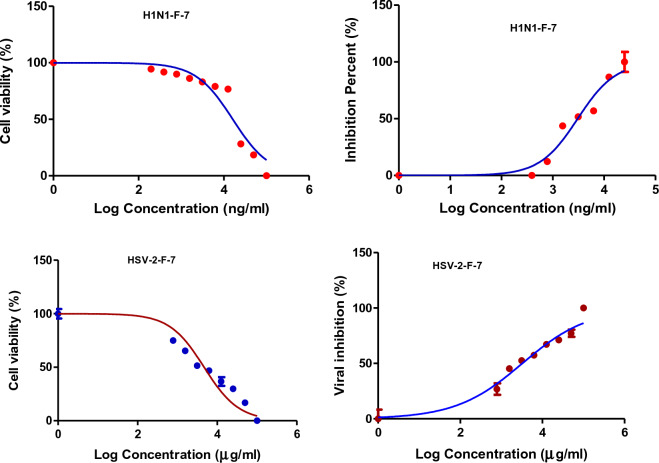
The effects of CH_2_CI_2_ fraction of *J. spinosa roots* (F-7) on the viral inhibition in H1N1 and HSV-2 cell lines.

The antiviral activities of CH_2_Cl_2_ fractions **F-6** and **F-7** of *J. variegata* and *J. spinosa* roots are presented in Table 4. These fractions showed the most significant activities against Human Herpes Simplex Type 2 (HSV-2) and Human Influenza (H1N1) cell lines (IC_50_ 4.88, 4.24, 3.4, and 3.06 μg/mL, respectively), whereas methanol extracts **Ext-3** and **Ext-5** of *J. vriegata* and *J. spinosa* roots were less active (IC_50_ > 50 μg/mL) as shown in Figs. 7, 8, 9, 10, whereas acyclovir showed IC_50_ of 83.19 µg/ml against HSV-2 cell line and ribavirin showed IC_50_ of 52.40 µg/ml against H1N1 cell line. **F-6** and **F-7** factions showed greater activities than other extracts when compared to standard drugs, with in vitro antiviral activities ranging from 3.06 to 4.88 μg/ mL. The determination of selectivity indices for the extracts and fractions revealed significant differences. The extracts Ext-3 and Ext-5, with IC_50_ values greater than 50 μg/mL, exhibited weak selectivity, ranging from SI 0.2 to 1.71. On the other hand, F-6 demonstrated high selectivity in terms of antiviral activity against HSV-2 (SI 5.9) and H1N1 (SI 3.4). Similarly, F-7 displayed good selectivity against H1N1 (SI 5.2). Both Fractions (F-6 and F-7) showed higher SI than each of acyclovir and ribavirin (Table 4).

**Table 4:** Antiviral activity of *J. variegata and J. spinosa* various MeOH extracts (Ext-3–5) and CH_2_Cl_2_ fractions (F-6 & F-7) against Human Herpes simplex type 2 (HSV-2) and Human Influenza (H1N1) cell lines.

Fraction /cell line	Human herpes simplex type 2 (HSV-2)			Human influenza (H1N1)		
	CC_50_ (µg/mL)	IC_50_ (µg/mL)	SI CC50/IC50	CC_50_ (µg/mL)	IC_50_ (µg/mL)	SI CC50/IC50
Ext-3 (MeOH of roots of *J. variegata*)	172.75 ± 29.25	101.23 ± 39.41	1.7	9.35 ± 25.82	51.29 ± 35.28	0.2
Ext-5 (MeOH of roots of *J. spinosa*)	52.04 ± 31.02	68.83 ± 33.63	0.76	47.68 ± 32.34	27.92 ± 31.84	1.71
F-6 (CH_2_Cl_2_ of roots of *J. variegata*)	29.14 ± 30.76	4.88 ± 31.04	5.9	14.50 ± 35.32	4.24 ± 37.24	3.4
F-7 (CH_2_Cl_2_ of roots of *J. spinosa*)	4.68 ± 30.56	3.24 ± 29.26	1.4	16.18 ± 34.93	3.06 ± 37.90	5.2
Acyclovir	416.35 ± 30.17	83.19 ± 31.44	5.0	-	-	-
Ribavirin	-	-	-	66.54 ± 29.31	52.40 ± 27.11	1.3

Data is represented as mean ± standard deviation; SI: Selectivity index.

### Biological findings

Cancer is a disease caused by the unregulated division of aberrant cells in a single location of the body, and it has the potential to infiltrate surrounding tissues^51^. There are about 200 different forms of cancer, with the most prevalent being breast, colon, lung, and prostate cancer. Brain tumors, carcinomas, leukaemias, lymphomas, and sarcomas are the five forms of cancer. Cancer is predicted to impact one in every two persons in the UK over their lifetime, and it is currently the world's second biggest cause of death^52^.

In both the treatment and prevention of cancer, natural therapies have been extremely important. Vincristine in 1963 and vinblastine in 1965, the vinca alkaloids isolated from *Catharanthus spp.* (Madagascar periwinkle plants) growing in the Philippines and Jamaica, were the first naturally derived anticancer drugs approved by the Food and Drug Administration (FDA) in the United States^53^, the most prevalent genital cancer is cervical cancer, which is also one of the main reasons why women die. It accounts for about 12% of all female malignancies worldwide, particularly in underdeveloped nations^54^.

The increasing number of cancer-related fatalities has prompted researchers to search for novel anticancer medications. Synthetic drug-based chemotherapeutic treatments have been used to treat cancer, but their usage has been limited by their high cost and potential for toxicity. The study of plant-based anticancer drugs is now advancing rapidly. According to a recent assessment, the majority of secondary metabolites isolated from numerous plant families have shown potential for development as anticancer medicines^55,56^. According to the WHO, natural drugs have the potential to treat between 65 and 80% of human diseases. Natural products are less expensive than synthetic drugs and have fewer side effects, which has led to an increase in the use of medicines derived from natural products^57^.

The possible cytotoxic properties of *J. variegata* and *J. spinosa,* two well-known bioactive medicinal herbs, were therefore examined in this work, as well as their potential impact on the cytotoxic profile of MCF-7, HEPG2, and A549 cell lines. To the best of our knowledge, no in vivo or in vitro studies of *J. variegata* and *J. spinosa's* anticancer and antiviral activities in various types of malignancies have been published before. This is the initial study of *J. variegata* and *J. spinosa's* effects on cancerous and viral cells.

## Conclusions

In conclusion, our study demonstrated the promising cytotoxicity and antiviral activities of various extracts and fractions derived from *J. variegata* and *J. spinosa*. These beneficial effects can be attributed to the high content of flavonoids and terpenoids present in these extracts and fractions.

Metabolomics profiling of *J. variegata and J. spinosa* revealed the presence of numerous compounds with well-established cytotoxic and antiviral activities. Among them, 14 diterpene compounds such as jatrophone, ferrugenol, curcusone, multifidone and 11 Flavonoids such as taxifolin, vitexin, and quercetin. These findings highlight the potential of *J. variegata* and *J. spinosa* extracts and fractions as a source of bioactive compounds for the development of cytotoxic and antiviral agents.

Overall, our results underscore the importance of exploring phytoconstituents derived from *J. variegata* and *J. spinosa,* as they hold promise for the development of novel therapeutic interventions against cancer and viral infections. Further research and investigations are warranted to elucidate the mechanisms of action and evaluate the potential clinical applications of these bioactive compounds.

### Supplementary Information

Supplementary Information.

## Data Availability

All data generated or analyzed during this study are included in this published article [and its supplementary information files].
