# Supplementary material for: Cytotoxic and antiviral activities of Jatropha variegata and Jatropha spinosa in relation to their metabolite profile
Source: Sci Rep. 2024 Feb 28;14:4846. doi: 10.1038/s41598-024-55196-1 (PMC10902333; doi:10.1038/s41598-024-55196-1)
Supplement: Supplementary file 1 — Supplementary Information. [file 41598_2024_55196_MOESM1_ESM.docx]

**Cytotoxic and Antiviral Activities of *Jatropha variegata* and *Jatropha spinosa* in Relation to their Metabolite Profile**

Khawlah Shari^1^, Osama G. Mohamed^1, 2^, Khaled M. Meselhy^1^, Ashootosh Tripathi^2, 3^, Amal E. Khaleel^1^, Essam Abdel-Sattar^1, *^, Rania A. El Gedaily^1^

^1^Pharmacognosy Department, Faculty of Pharmacy, Cairo University, Kasr el Aini St., Cairo 11562, Egypt

^2^Natural Products Discovery Core, Life Sciences Institute, University of Michigan, Ann Arbor, MI 48109, USA

^3^Department of Medicinal Chemistry, College of Pharmacy, University of Michigan, Ann Arbor, MI 48109, USA

*****Corresponding author:

Essam Abdel-Sattar

essam.abdelsattar@pharma.cu.edu.eg; Tel.: [+2023639307](Tel:23639307); Fax: +2023628426,

Cell phone: +201065847211

**Content**

**Fig. S1.** The effect of methanol extracts (Ext-1, 2, 3, 4, and 5) of J. variegata and J. spinosa on the cytotoxicity in MCF-7, HEPG-2 and A549 cell lines.

**Fig. S2.** The effect of CH_2_Cl_2_ fractions (F-6 and F-7) of J. variegata and J. spinosa on the cytotoxicity in MCF-7, HEPG-2 and A549 cell lines.

**Fig. S3.** The effect of methanol extracts (Ext-3 and Ext-5) of J. variegata and J. spinosa roots on the viral inhibition in H1N1 and HSV-2 cell lines.

**Fig. S4.** The effect of CH_2_Cl_2_ fractions (F-6 and F-7) of J. variegata and J. spinosa roots on the viral inhibition in H1N1 and HSV-2 cell lines.

**Fig. S5.** Base peak chromatograms (BPC) of the MeOH extracts of J. variegata and J. spinosa roots in negative (A-B) and positive ioniozation modes and (C-D), respectively.

**Fig. S1.** The effect of methanol extracts (Ext-1, 2, 3, 4, and 5) of J. variegata and J. spinosa on the cytotoxicity in MCF-7, HEPG-2 and A549 cell lines.

**Fig. S2.** The effect of CH_2_Cl_2_ fractions (F-6 and F-7) of J. variegata and J. spinosa on the cytotoxicity in MCF-7, HEPG-2 and A549 cell lines.

**Fig. S3.** The effect of methanol extracts (Ext-3 and Ext-5) of J. variegata and J. spinosa roots on the viral inhibition in H1N1 and HSV-2 cell lines.

**Fig. S4.** The effect of CH_2_Cl_2_ fractions (F-6 and F-7) of J. variegata and J. spinosa roots on the viral inhibition in H1N1 and HSV-2 cell lines.


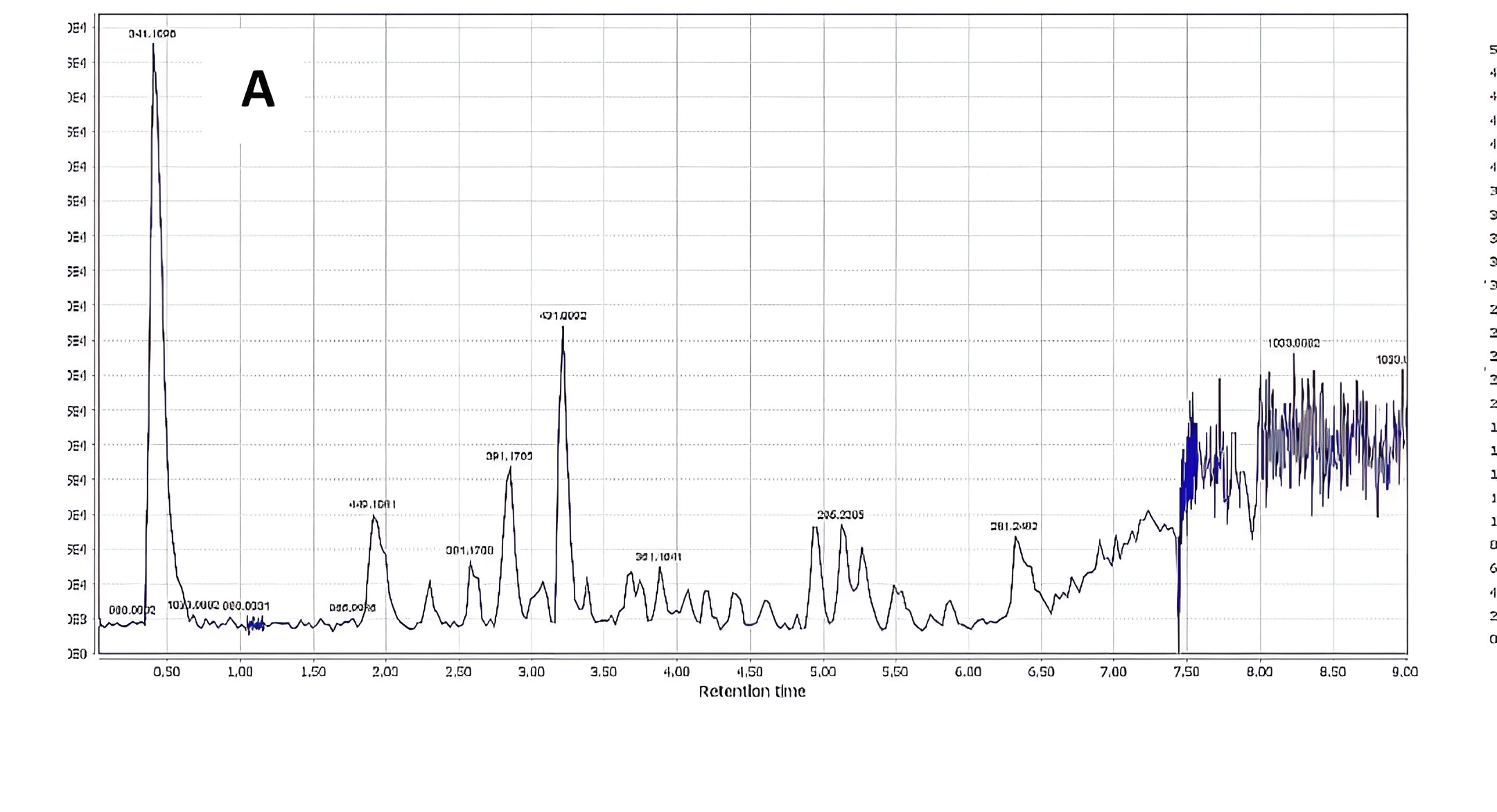

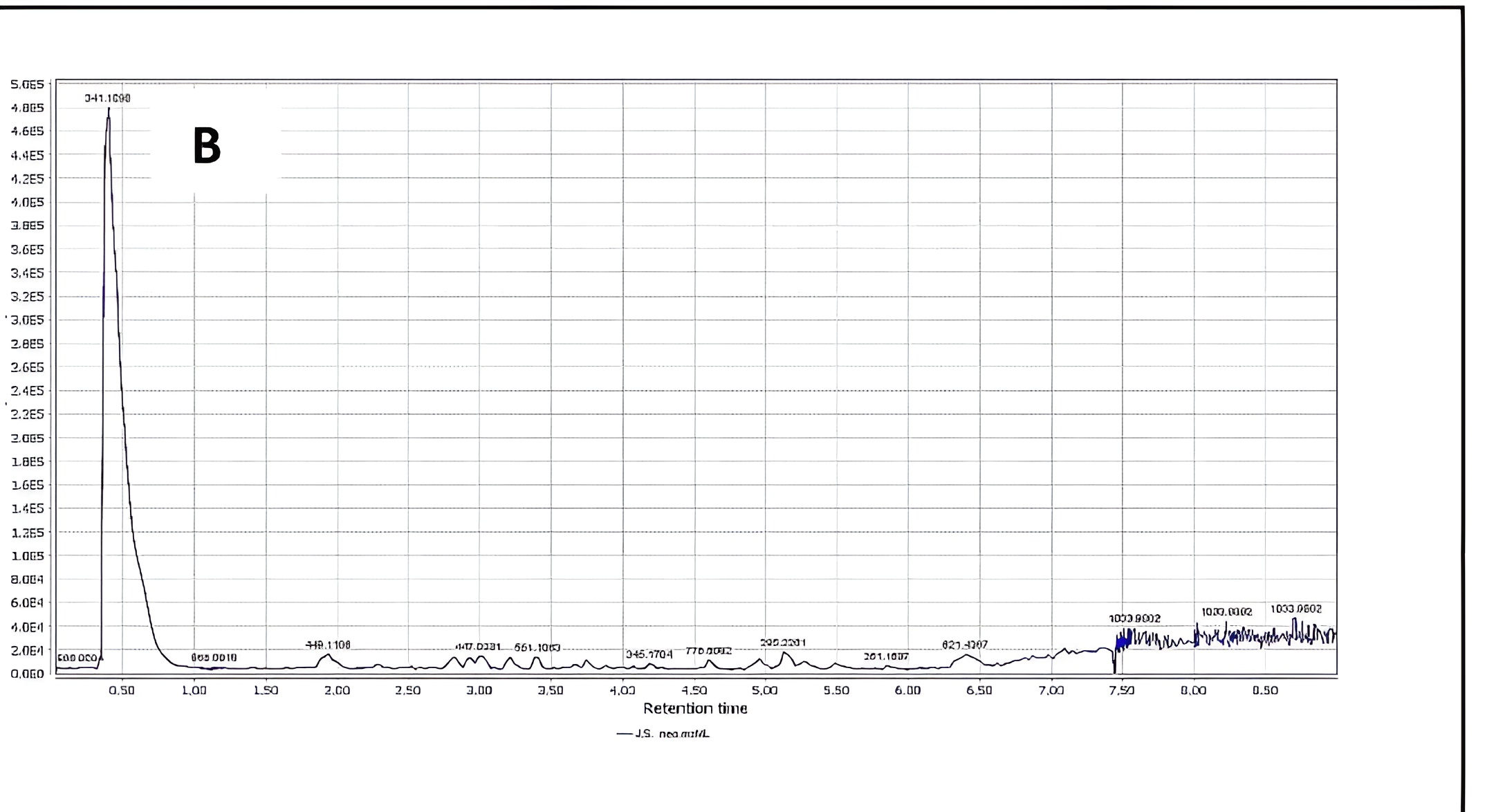


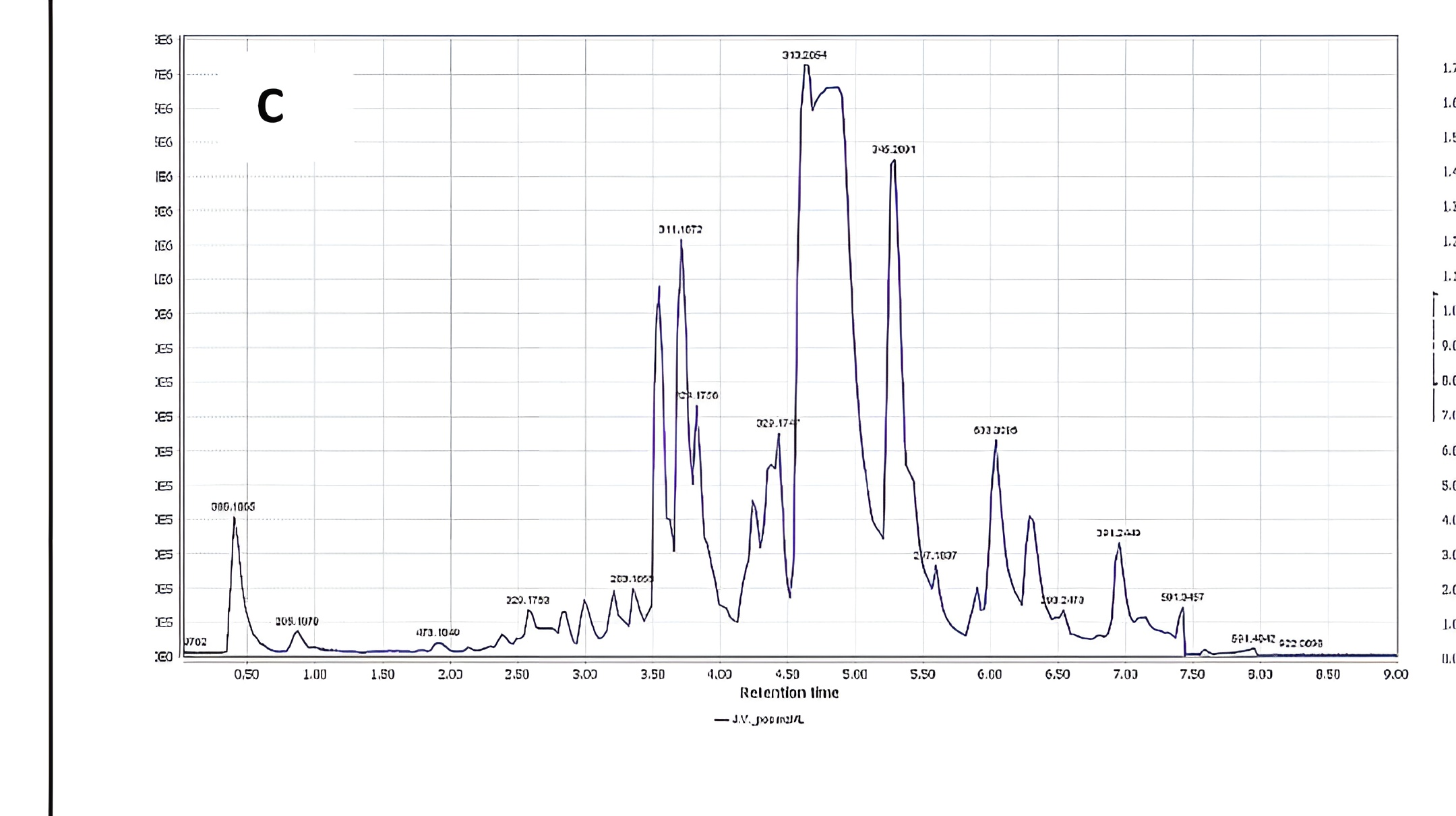

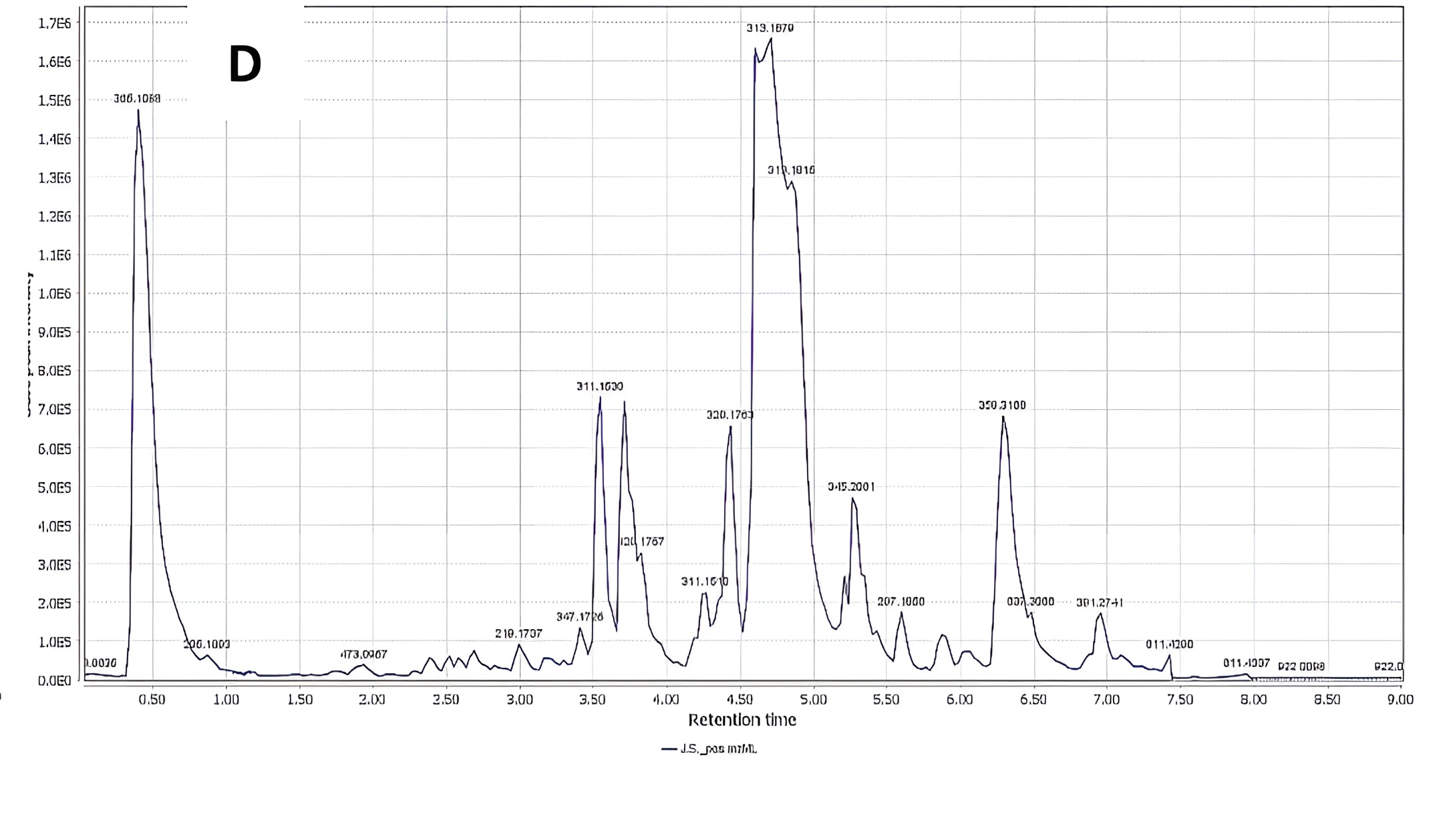


**Fig. S5. Base peak chromatograms (BPC) of the MeOH extracts of *J. variegata* and *J. spinosa* roots in negative (A-B) and positive ioniozation modes and (C-D), respectively.**
